# Characterizing Vericiguat Treatment in Heart Failure: A Multicenter Real-World Study in China

**DOI:** 10.31083/j.rcm2512427

**Published:** 2024-11-29

**Authors:** Qi Wang, Guangchuan Wang, Jiecheng Peng, Jingjing Li, Changlin Ju, Lingxin Pan, Zhiwei Xu, Jun Qian, Zhiquan Liu, Guohong Wu, Xueping Wei, Ji Yan, Xuejun Xiang, Kangyu Chen, on behalf of the HCDCG

**Affiliations:** ^1^Department of Cardiology, The First Affiliated Hospital of USTC, Division of Life Sciences and Medicine, University of Science and Technology of China, 230001 Hefei, Anhui, China; ^2^Department of Cardiology, Huaibei People’s Hospital, 235000 Huaibei, Anhui, China; ^3^Department of Cardiology, The First People’s Hospital of Anqing Affiliated to Anhui Medical University, 246004 Anqing, Anhui, China; ^4^Department of Cardiology, The Third People’s Hospital of Bengbu, 233000 Bengbu, Anhui, China; ^5^Department of Cardiology, The First Affiliated Hospital of Wannan Medical College, 241001 Wuhu, Anhui, China; ^6^Department of Cardiology, People’s Hospital of Tongling, 244000 Tongling, Anhui, China; ^7^Department of Cardiology, Anhui No.2 Provincial People’s Hospital, 230041 Hefei, Anhui, China; ^8^Department of Cardiology, Maanshan General Hospital of Ranger-Duree Healthcare, 243071 Maanshan, Anhui, China; ^9^Department of Cardiology, Anqing Municipal Hospital, 246004 Anqing, Anhui, China

**Keywords:** heart failure, vericiguat, real-world study

## Abstract

**Background:**

Real-world data on the clinical benefit of vericiguat are currently limited. This multicenter, real-world study was conducted to evaluate the clinical characteristics and therapeutic effects of vericiguat in real-world settings.

**Methods:**

This study analyzed heart failure (HF) patients who initiated vericiguat treatment from September 2022 to August 2023 across nine hospitals in the Anhui Province, China. The clinical data were retrospectively collected and cases were prospectively followed to assess changes from baseline in N-terminal pro-B type natriuretic peptide (NT-proBNP) at 12 months. Baseline characteristics were compared with those in the VICTORIA trial.

**Results:**

Of the 285 patients enrolled, the mean age was 64.8 ± 12.9 years. Of these, 22.8% were female, and 94.7% were classified as New York Heart Association class III–IV. Additionally, 66.4% had a reduced ejection fraction with a median NT-proBNP level of 2915 pg/mL. Vericiguat therapy was initiated during hospitalization in 223 patients (78.2%), with 105 (37.1%) receiving quadruple anti-HF therapy. Only 44.9% met the VICTORIA trial inclusion criteria.

**Conclusions:**

In this multicenter, real-world study of vericiguat in Anhui Province, only 44.9% of vericiguat users met the inclusion criteria for the VICTORIA trial. This suggests that vericiguat is being applied more broadly by physicians to treat HF in real clinical settings.

## 1. Introduction

Heart Failure (HF) is a life-threatening clinical syndrome associated with high morbidity and mortality, diminished quality of life, and significant healthcare expenses, affecting an estimated 64 million individuals worldwide [1]. In recent years, important advances have been made in the pharmacological treatment of HF. Angiotensin neprilysin inhibitors (ARNIs), for instance, have shown superiority over enalapril in reducing the risk of cardiovascular death or hospitalization in patients experiencing heart failure with reduced ejection fraction (HFrEF) [2]. Additionally, sodium glucose cotransporter 2 inhibitors (SGLT2Is) have been demonstrated to improve outcomes across the full spectrum of ejection fractions by modulating metabolic pathways [3, 4, 5, 6]. Based on these developments, current guidelines from the ESC and AHA/ACC/HFSA strongly supports a quadruple therapy regimen comprising the above novel agents combined with conventional beta-blockers and mineralocorticoid receptor antagonists (MRAs) for managing HF [7, 8].

Vericiguat is a novel soluble guanylate cyclase (sGC) inducer that enhances endothelial function in HF patients by restoring the nitric oxide (NO)-sGC-cyclic guanosine monophosphate (cGMP) pathway, conferring cardiac and renal benefits [9]. To date, its clinical utility has been supported by only a single randomized controlled trial (RCT), the VICTORIA trial, which demonstrated that vericiguat reduces the risk of cardiovascular death or hospitalization in HF patients with a left ventricular ejection fraction (LVEF) below 45% [10]. Apart from this, only a single-center, retrospective study involving 28 HF patients receiving vericiguat treatment [11]. Therefore, there is a pressing need for large multicenter, real-world studies to further assess the current status of the clinical use and effectiveness of vericiguat in a broader patient population.

To provide further evidence for vericiguat use in the real world, a multicenter study was carried out in Anhui Province, China. The aim was to evaluate the clinical characteristics of patients treated with vericiguat and to assess the treatment’s effectiveness, thereby informing clinical practice guidelines. This paper presents the design of a multicenter study along with a preliminary analysis of the baseline features of the included patients.

## 2. Methods

### 2.1 Study Population and Protocol

**Fig. 1.**
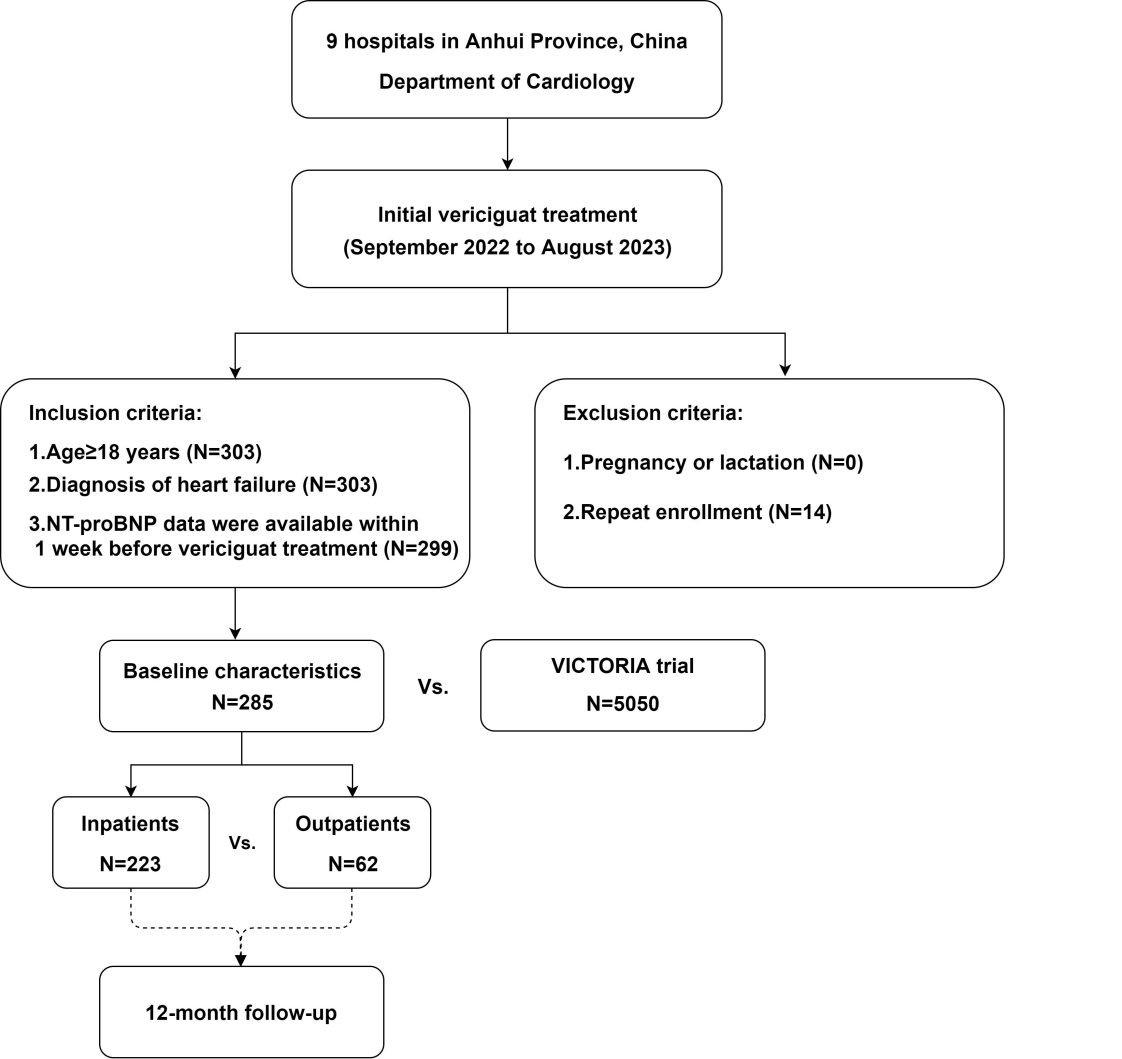
** Design and enrollment criteria for the multicenter vericiguat treatment study**. This flowchart outlines the recruitment process in the context of inclusion and exclusion criteria. NT-proBNP, N-terminal pro-B type natriuretic peptide.

The study population consisted of HF patients who initiated vericiguat treatment from September 2022 to August 2023, as documented in the cardiology departments of nine hospitals (both outpatient and inpatient settings) in Anhui Province (**Supplementary Table 1**). Inclusion criteria were: (1) ≥18 years old; (2) HF diagnosis in accordance with the Chinese Guidelines for the Diagnosis and Treatment of HF 2018 [12]; (3) N-terminal pro-B type natriuretic peptide (NT-proBNP) data available within 1 week prior to starting vericiguat treatment. Exclusion criteria were: (1) pregnancy or lactation; (2) repeat outpatient or inpatient enrolment. The use of vericiguat mainly follows the indications approved in China. Indications: Vericiguat is indicated for the treatment of symptomatic chronic HF in adult patients with reduced ejection fraction (EF <45%) who are stabilized after a recent decompensation event requiring intravenous therapy. Contraindications: (1) Concomitant use of other sGC stimulators; (2) Use in pregnant women. The primary study endpoint was the change from baseline of NT-proBNP at 12 months. Secondary endpoints included changes in echocardiographic parameters (LVEF, left ventricular end-diastolic volume, and left ventricular end-systolic volume) from baseline at 12 months. The clinical data of the patients enrolled before August 2023 were retrospectively collected, and the cases were prospectively followed up according to the time of vericiguat treatment. Age, sex, duration of HF, etiology, comorbidities, New York Heart Association (NYHA) class, NT-proBNP levels, renal function, electrolytes, echocardiography findings, and HF treatment drugs or devices for the patients were collected. HF hospitalizations and deaths were recorded through outpatient and telephone follow-up. The study flowchart is shown in Fig. 1.

This paper analyses baseline data of patients who initiated vericiguat treatment, categorizing them by the type of visit—outpatient versus inpatient. It compares clinical characteristics between these groups and assesses the proportion of patients meeting the VICTORIA trial’s selection criteria. Differences in clinical features between study participants and those of the VICTORIA trial are also examined.

### 2.2 Statistical Analysis

Continuous variables were expressed as mean ± standard deviation for normally distributed data and median (1st and 3rd quartiles) for skewed data. Student’s *t* test were used for between-group comparisons of continuous variables while the Mann-Whitney U test was applied to skewed data. Categorical variables were expressed as frequency (percentage) and compared by the χ^2^ test or Fisher’s exact test, as appropriate. A two-tailed *p* < 0.05 was considered statistically significant. All analyses were performed with SPSS version 22.0 (IBM Corp., Armonk, NY, USA).

## 3. Results

Between September 2022 and August 2023, 285 HF patients were administered vericiguat in the cardiology departments of nine hospitals in Anhui Province. The mean age of participants was 64.8 ± 12.9 years, and 22.8% of cases in the cohort were female. The median duration of HF was 1.8 years (interquartile range [IQR]: 0.6, 5.1), with 66.4% of patients diagnosed with HFrEF and 7% experiencing HF with preserved ejection fraction (HFpEF). The majority of cases (94.7%) were classified as NYHA class III–IV, and the median NT-proBNP levels were 2915 pg/mL. The common causes of HF included coronary artery disease (36.8%) and hypertension (34.0%). Pharmacologically, most patients were administered a mineralocorticoid receptor antagonist (MRA) (84.8%), a beta-blocker (74.3%), and a renin-angiotensin system inhibitor (RASI)/ARNI (3.9%/68.3%), with a median ARNI dose of 100 mg. More than half (52.7%) of the patients were administered the above triple combination, while 68.7% were also administered SGLT2Is, resulting in the use of quadruple anti-HF drugs by 37.1% cases. Device use for HF was low, with 8.4% of patients undergoing cardiac resynchronization therapy (CRT) and 11.2% receiving implantable cardioverter-defibrillator (ICD). Detailed baseline features of the study population are shown in Table 1.

**Table 1.** **Baseline clinical characteristics of patients in the multicenter vericiguat study versus study versus the VICTORIA trial**.

Characteristics		Overall (N = 285)	VICTORIA trial (N = 5050)
Age, y		64.8 ± 12.9	67.3 ± 12.2
Female sex		65 (22.8)	1208 (23.9)
Hospitalization for HF *		154/187 (82.4)	4249 (84.1)
Body mass index, kg/m^2^		24.1 ± 3.6	27.8 ± 5.9
New York Heart Association class			
	I	3 (1.1)	2/5046 (<0.1)
	II	12 (4.2)	2975/5046 (59.0)
	III	157 (55.1)	2003/5046 (39.7)
	IV	113 (39.6)	66/5046 (1.3)
HF duration, y		3.6 ± 4.4	4.8 ± 5.4
		1.8 (0.6, 5.1)	
Left ventricular ejection fraction		34.9 ± 10.4	28.9 ± 8.3
Left ventricular ejection fraction <40%		180/271 (66.4)	4316 (85.7)
Systolic blood pressure, mm Hg		124.0 ± 22.1	121.4 ± 15.7
Diastolic blood pressure, mm Hg		77.9 ± 15.6	72.8 ± 11.0
Heart rate, beats/min		83.5 ± 19.1	73.1 ± 13.0
Atrial fibrillation or atrial flutter		53 (18.6)	2660/5048 (52.7)
Diabetes mellitus		33 (11.6)	2369/5048 (46.9)
Hypertension		97 (34.0)	3995/5048 (79.1)
Stroke		29 (10.2)	578/5048 (11.5)
CAD		105 (36.8)	2944/5048 (58.3)
Standard of care treatment			
	ACE inhibitor or ARB	11/284 (3.9)	3700/5040 (73.4)
	Angiotensin receptor–neprilysin inhibitor	194/284 (68.3)	731/5040 (14.5)
	Beta blocker	211/284 (74.3)	4691/5040 (93.1)
	MRA	240/283 (84.8)	3545/5040 (70.3)
	Triple therapy	149/283 (52.7)	3009/5040 (59.7)
	SGLT2 inhibitor	195/284 (68.7)	Unknown
	Quadruple therapy	105/283 (37.1)	Unknown
	ICD	32 (11.2)	1399/5040 (27.8)
	Biventricular pacemaker	24 (8.4)	739/5040 (14.7)
Laboratory results			
	Hemoglobin, g/dL	13.3 ± 2.2	13.4 ± 1.9
	Sodium, mEq/L	140.5 ± 4.1	139.9 ± 3.4
	Potassium, mEq/L	4.2 ± 0.7	4.5 ± 0.5
	Estimated GFR, mL/(min·1.73 m^2^)	59.7 (43.7, 88.2)	58.4 (41.2, 77.1)
Estimated GFR categories			
	≤30	31/241 (12.9)	506/4959 (10.2)
	>30 to ≤60	93/241 (38.6)	2118/4959 (42.7)
	>60	117/241 (48.5)	2335/4959 (47.1)
	NT-proBNP, pg/mL	2915.0 (1074.0, 9020.1)	2816.0 (1556.0, 5314.0)

*In the VICTORIA trial it was hospitalization for HF in the previous 6 months. ACE, angiotensin converting enzyme; ARB, angiotensin receptor blocker; CAD, coronary artery disease; GFR, glomerular filtration rate; ICD, implantable cardioverter-defibrillator; MRA, mineralocorticoid receptor antagonist; NT-proBNP, N-terminal pro-B-type natriuretic peptide; SGLT2, sodium glucose cotransporter 2; y, year; HF, heart failure.

The majority of patients (78.2%) initiated vericiguat therapy during hospitalization, with a median hospitalization of 9 days (IQR: 6, 13). The starting dose of vericiguat in 96.1% of patients was 2.5 mg once daily. In comparison to outpatients, inpatients exhibited higher blood pressure, faster heart rate, and poorer renal function. A higher proportion of inpatients were classified as NYHA class IV and presented with elevated NT-proBNP levels. Although the use of triple anti-HF drugs was more prevalent among inpatients compared with outpatients, the utilization of SGLT2I was lower, resulting in similar rates of quadruple anti-HF drug therapy between the two groups. In addition, CRT and ICD implantation rates were lower in inpatients compared with outpatients. Detailed comparisons of these baseline features between the two groups are shown in Table 2.

**Table 2.** **Comparitive baseline characteristics of inpatients versus outpatients initiating vericiguat treatment**.

Characteristics		Outpatients (N = 62)	Inpatients (N = 223)	*p* value
Age, y		63.5 ± 11.6	65.2 ± 13.3	0.364
Female sex		14 (22.6)	51 (22.9)	0.962
Hospitalization for HF		38/50 (76.0)	116/137 (84.7)	0.169
Body mass index, kg/m^2^		24.1 ± 3.5	24.1 ± 3.7	0.948
New York Heart Association class				0.019
	I	0 (0.0)	3 (1.3)	
	II	2 (3.2)	10 (4.5)	
	III	45 (72.6)	112 (50.2)	
	IV	15 (24.2)	98 (43.9)	
HF duration, y		5.2 ± 6.0	3.0 ± 3.4	0.074
		3.2 (0.6, 8.1)	1.6 (0.7, 4.2)	
Left ventricular ejection fraction		34.9 ± 9.4	34.9 ± 10.7	0.989
Left ventricular ejection fraction <40%		37/55 (67.3)	143/216 (66.2)	0.881
Systolic blood pressure, mm Hg		116.2 ± 17.4	126.2 ± 22.8	<0.001
Diastolic blood pressure, mm Hg		72.6 ± 12.4	79.4 ± 16.1	0.002
Heart rate, beats/min		76.1 ± 15.5	85.6 ± 19.5	<0.001
Atrial fibrillation or atrial flutter		11 (17.7)	42 (18.8)	0.845
Diabetes mellitus		6 (9.7)	27 (12.1)	0.597
Hypertension		19 (30.6)	78 (35.0)	0.524
Stroke		6 (9.7)	23 (10.3)	0.883
CAD		19 (30.6)	86 (38.6)	0.253
Standard of care treatment				
	ACE inhibitor or ARB	0 (0.0)	11/222 (5.0)	0.129
	Angiotensin receptor–neprilysin inhibitor	36 (58.1)	158/222 (71.2)	0.050
	Beta blocker	40 (64.5)	171/222 (77.0)	0.046
	MRA	42 (67.7)	198/221 (89.6)	<0.001
	Triple therapy	24 (38.7)	125/221 (56.6)	0.013
	SGLT2 inhibitor	49 (79.0)	146/222 (65.8)	0.046
	Quadruple therapy	23 (37.1)	82/221 (37.1)	0.999
	ICD	13 (21.0)	19 (8.5)	0.006
	Biventricular pacemaker	12 (19.4)	12 (5.4)	<0.001
Laboratory results				
	Hemoglobin, g/dL	13.7 ± 2.2	13.2 ± 2.1	0.166
	Sodium, mEq/L	140.2 ± 2.8	140.5 ± 4.3	0.598
	Potassium, mEq/L	4.2 ± 0.4	4.2 ± 0.7	0.287
	Estimated GFR, mL/(min·1.73 m^2^)	59.7 (43.7, 88.2)	58.4 (41.2, 77.1)	0.040
	NT-proBNP, pg/mL	1081.0 (594.0, 2380.0)	3996.5 (1465.5, 10,572.0)	<0.001

ACE, angiotensin converting enzyme; ARB, angiotensin receptor blocker; CAD, coronary artery disease; GFR, glomerular filtration rate; HF, heart failure; ICD, implantable cardioverter-defibrillator; MRA, mineralocorticoid receptor antagonist; NT-proBNP, N-terminal pro-B-type natriuretic peptide; SGLT2, sodium glucose cotransporter 2; y, year.

When comparing the selection criteria of the current study to the VICTORIA trial (Table 3), only 44.9% of current patients met the VICTORIA trial inclusion criteria, with a compliance rate of 50.0% for inpatients. An analysis of the exclusion variables showed that 14.4% of cases were excluded based on systolic blood pressure (SBP) and 1.2% based on renal function, per VICTORIA’s exclusion criteria. Compared to the VICTORIA trial participants, patients in this study exhibited lower body mass index, a shorter duration of HF, higher baseline LVEF, and lower rates of coronary artery disease, hypertension, atrial fibrillation or flutter, and diabetes mellitus rates. Moreover, there was a higher proportion of NYHA class III–IV cases in this study. Treatment differences were notable, unlike the VICTORIA trial, ARNI largely replaced RASI for HF treatment in the present study, with added use of SGLT2Is. Use of MRA was higher in this study compared with VICTORIA, while use of beta blockers was lower. Additionally, CRT and ICD implantation rates were lower in this study compared with the VICTORIA trial.

**Table 3.** **Fulfilment of VICTORIA trial inclusion and key exclusion criteria**.

		Total (N = 285)	Inpatients (N = 223)
Inclusion criteria			
	Informed consent (assumed 100%)	285/285 (100.0)	223/223 (100.0)
	Age ≥18 years	284/284 (100.0)	223/223 (100.0)
	Chronic HF (HF duration ≥6 months)	149/185 (80.5)	108/135 (80.0)
	NYHA class II–IV	282/285 (98.9)	220/223 (98.7)
	Prior HF hospitalization within 6 months*	154/187 (82.4)	116/137 (84.7)
	NT-proBNP criterion	207/273 (75.8)	182/218 (83.5)
	Left ventricular ejection fraction <45%	227/271 (83.8)	181/216 (83.8)
	Is not of reproductive potential (assumed 100%)	285/285 (100.0)	223/223 (100.0)
	Trial eligibility, only inclusion criteria	75/167 (44.9%)	63/126 (50.0)
Key exclusion criteria			
	SBP <100 mmHg at baseline	41/285 (14.4)	27/223 (12.1)
	Estimated GFR <15 mL/min/1.73 m^2^ at baseline	3/241 (1.2)	3/201 (1.5)

^*^This study was defined as a previous history of hospitalization for HF. GFR, glomerular filtration rate; HF, heart failure; NT-proBNP, N-terminal pro-B-type natriuretic peptide; NYHA, New York Heart Association class; SBP, systolic blood pressure.

## 4. Discussion

This multicenter real-world study conducted in Anhui Province, China, analyzed the baseline characteristics of 285 HF patients who initiated vericiguat treatment. A significant proportion (94.7%) of these patients were classified as NYHA class III–IV, and 66.4% were diagnosed with HFrEF. More than half of the patients were administered triple anti-HF drugs, and 68.7% received SGLT2I treatment. However, only 44.9% of the patients in the current study met the inclusion criteria of the VICTORIA trial. When compared with the VICTORIA cohort, the patients in this study exhibited lower rates of coronary artery disease, hypertension, atrial fibrillation or flutter, and diabetes mellitus. While NT-proBNP levels were similar, this study’s cohort showed higher LVEF values and a greater proportion of patients in NYHA class III–IV.

Since vericiguat has emerged as a novel anti-HF drug, it is critical to select the appropriate patients to assess the real-world outcomes. Nguyen *et al*. [13] investigated eligibility for vericiguat among 23,573 HFrEF patients in the Swedish HF registry, finding that only 21.4% met the VICTORIA trial criteria, compared to 47.4% who were eligible based on guidelines and the drug’s label. Similarly, data from the Get With The Guidelines-Heart Failure (GWTG-HF) registry, which included 241,057 patients with LVEF below 45%, suggested that nearly 4 in 10 patients would be eligible for vericiguat per VICTORIA’s selection criteria, and 9 in 10 patients would be eligible based on the Food and Drug Administration (FDA) label [14]. In the Korean Acute HF registry, 58% of patients met the trial criteria for vericiguat [15]. In contrast, this study found that just under half of all patients administered vericiguat in Anhui Province, China, fulfilled the VICTORIA criteria, suggesting a more aggressive use of this medication by physicians real clinical settings.

Significant discrepancies between patient characteristics in clinical trials and real-world practices are well-documented, largely due to the stringent selection criteria of randomized controlled trials. Due to its minimal impact on blood pressure [16], vericiguat is often prescribed early in the course of treatment for individuals with low blood pressure. In the current study, 14.4% of patients had baseline SBP below 100 mmHg, and nearly 20% had been diagnosed with HF for less than six months. The proportion of patients in NYHA class III–IV was higher in this study compared to the VICTORIA trial, while the median NT-proBNP levels were similar [17]. However, only 75.8% of patients met VICTORIA’s inclusion criteria based on NT-proBNP levels, with most discrepancies occurring in outpatients. Notably, outpatients with high NT-proBNP levels, especially those previously administered quadruple anti-HF drugs, were often prescribed vericiguat, despite not meeting the VICTORIA criteria [18]. It is noteworthy that 12.9% of patients in this study had an eGFR below 30 mL/min/1.73 m^2^, including three individuals with an eGFR below 15 mL/min/1.73 m^2^. These differences in patient features underscore that in clinical setting, physicians tend to prescribe vericiguat early for specific HF cases, particularly when quadruple anti-HF drugs prove ineffective or are contraindicated.

It is important to consider the interactions between vericiguat and other anti-HF drugs, such as SGLT2Is and ARNIs, which are unclear. Currently, SGLT2Is are guideline-recommended for HFrEF treatment [7], and were used by 68.7% of patients in this study, although they were not included in the VICTORIA trial. Although both drug groups have distinct mechanisms in HF treatment [19, 20], the potential contribution of SGLT2Is on the efficacy of vericiguat cannot be ignored. One study suggested ARNIs do not affect vericiguat efficacy [21], however both drug categories interact with the natriuretic peptide system [22], warranting further exploration of their combined effects.

This study is subject to several limitations. Firstly, the sample size was relatively small, and the baseline data were retrospectively collected, leading to instances of missing data (**Supplementary Table 2**). Secondly, due to gaps in the dataset, certain selection criteria from the VICTORIA trial could not be accurately assessed, including the date of last HF hospitalization, outpatient intravenous diuretic therapy, and reproductive potential as well as other factors. Additionally, our evaluation of guideline-directed medical therapy (GDMT) criteria did not account for individual drug dosages or adherence, owing to the unavailability of these data. Finally, the real world effectiveness of vericiguat requires further exploration through continued follow-up in this study.

## 5. Conclusions

In this multicenter real-world study of vericiguat in China, only 44.9% of the participants met the VICTORIA inclusion criteria. The current study suggests that vericiguat may be more widely employed in clinical settings than is typical in clinical trials to treat HF. Further research is necessary to assess the effectiveness of vericiguat within the broader real-world HF population.

## Availability of Data and Materials

All data reported in this paper will be shared by the lead contact upon request.

## Supplementary Material

Supplementary material associated with this article can be found, in the online version, at https://doi.org/10.31083/j.rcm2512427.
