# Supplementary material for: Characterizing Vericiguat Treatment in Heart Failure: A Multicenter Real-World Study in China
Source: Rev Cardiovasc Med. 2024 Nov 29;25(12):427. doi: 10.31083/j.rcm2512427 (PMC11683721; doi:10.31083/j.rcm2512427)
Supplement: Supplementary file 1 [file 2153-8174-25-12-427-s1.pdf]

Baseline characteristics of heart failure patients with initiated vericiguat treatment: a multicenter real-world study in China

Supplementary Material

Table S1. List of hospitals participating in this study

Table S2. Missing data

Table S1. List of hospitals participating in this study

Hospital Name	Department	City
The First Affiliated Hospital of USTC	Cardiology	Hefei
Huaibei People's Hospital	Cardiology	Huaibei
The First People's Hospital of Anqing affiliated to Anhui Medical University	Cardiology	Anqing
The Third People's Hospital of Bengbu	Cardiology	Bengbu
The First Affiliated Hospital of Wannan Medical College	Cardiology	Wuhu
People’s Hospital of Tongling	Cardiology	Tongling
Anhui No.2 Provincial People's Hospital	Cardiology	Hefei
Maanshan General Hospital of Ranger-Duree Healthcare	Cardiology	Maanshan
Anqing Municipal Hospital	Cardiology	Anqing

Table S2. Missing data

Characteristics	Missing number	Missing rate (%)
Hospitalisation for heart failure	98	34.4
Body mass index, kg/m^2	97	34.0
Heart failure duration, y	99	34.7
Left ventricular ejection fraction	14	4.9
ACE inhibitor or ARB	1	0.35
Angiotensin receptor–neprilysin inhibitor	1	0.35
Beta blocker	1	0.35
MRA	2	0.70
SGLT2 inhibitor	1	0.35
Hemoglobin, g/dL	22	7.7
Sodium, mEq/L	27	9.5
Potassium, mEq/L	12	4.2
Estimated GFR, mL/(min·1.73 m^2)	44	15.4
NT-proBNP, pg/mL	12	4.2

ACE, angiotensin converting enzyme; ARB, angiotensin receptor blocker; GFR, glomerular filtration rate; MRA, mineralocorticoid receptor antagonist; NT-proBNP, N-terminal pro-B-type natriuretic peptide; SGLT2, Sodium glucose cotransporter 2.
